# Metformin use and the risk of bacterial pneumonia in patients with type 2 diabetes

**DOI:** 10.1038/s41598-022-07294-1

**Published:** 2022-02-28

**Authors:** Fu-Shun Yen, James Cheng-Chung Wei, Ying-Hsiu Shih, Chih-Cheng Hsu, Chii-Min Hwu

**Affiliations:** 1 Dr. Yen’s Clinic, Taoyüan, 271 Taiwan; 2 Division of Allergy, Immunology and Rheumatology, Chung Shan Medical University Hospital, Taichung City, 402 Taiwan; 3 Management Office for Health Data, China Medical University Hospital, Taichung, 404 Taiwan; 4 College of Medicine, China Medical University, Taichung City, 404 Taiwan; 5 Institute of Population Health Sciences, National Health Research Institutes, 35 Keyan Road, Zhunan, Miaoli County, 35053 Taiwan; 6 Ceneter for Geriatrics and Welfare Research, National Health Research Institutes, 35 Keyan Road, Zhunan, Miaoli County, 35053 Taiwan; 7 Department of Health Services Administration, China Medical University, Taichung, 404 Taiwan; 8 Department of Family Medicine, Min-Sheng General Hospital, Taoyüan, 330 Taiwan; 9 Faculty of Medicine, National Yang-Ming Chiao Tung University School of Medicine, No. 155, Sec. 2, Linong Street, Taipei, 11221 Taiwan; 10 Section of Endocrinology and Metabolism, Department of Medicine, Taipei Veterans General Hospital, Taipei, 11221 Taiwan

**Keywords:** Type 2 diabetes, Bacterial infection

## Abstract

Persons with type 2 diabetes (T2D) have neutrophil dysfunction with a higher risk of infection than those without diabetes. We conducted this study aiming to compare the risk of pneumonia between metformin use and nonuse in persons with T2D. We identified 49,012 propensity score-matched metformin users and nonusers from Taiwan’s National Health Insurance Research Database between January 1, 2000, and December 31, 2017. We used the Cox proportional hazards model to compare the risks of pneumonia and respiratory death. The mean (SD) age of the participants was 57.46 (12.88) years, and the mean follow-up time for metformin users and nonusers was 5.47 (3.71) years and 5.15 (3.87) years, respectively. Compared with the nonuse of metformin, the adjusted hazard ratios (95% CI) for metformin use in bacterial pneumonia, invasive mechanical ventilation, and respiratory cause of death were 0.89 (0.84–0.94), 0.77 (0.73–0.82), and 0.64 (0.56–0.74), respectively. A longer cumulative duration of metformin use had further lower adjusted hazard ratios in these risks compared with nonuse. In patients with T2D, metformin use was associated with significantly lower risks of bacterial pneumonia, invasive mechanical ventilation, and respiratory cause of death; moreover, longer metformin use duration was associated with lower hazard ratios of these risks.

## Introduction

Polymorphonuclear neutrophils adhere to vascular endothelium and transmigrate through blood vessels in response to chemotactic gradients to phagocytose and kill invading pathogens^1^. Reports suggest that persons with type 2 diabetes (T2D) have neutrophil dysfunction and disturbed cytokine production due to cumulated hyperglycemia and excess oxidative stress^2^, with a higher risk of infection than those without diabetes^3^. Studies have demonstrated that persons with T2D have a higher risk of the following than those without T2D: 3.0- to 4.3-fold for urinary tract infections, 1.8- to 2.0-fold for cellulitis, 1.2- to 2.6-fold for pneumonia, and 2.0- to 3.3-fold for sepsis^4^. People with T2D have a higher risk of developing comorbidities and vascular complications, and the presence of infection can increase morbidity and mortality^3,4^. The diabetes guidelines for infection are scarce^5^.

Persons with T2D may have pulmonary microangiopathy and impaired lung function^6^, with an increased risk of respiratory failure or death due to pneumonia^3^. According to Taiwan’s Diabetes Atlas report (2019), the odds ratios for pneumonia hospitalization in patients with T2D versus non-diabetes ranged from 2.86 to 2.93 between 2005 and 2014, with a significantly increasing trend^7^. Pneumonia constituted 3.52–7.27% of the leading cause of death in patients with T2D from 2005 to 2014, with a significantly rising trend^8^.

Metformin has long been considered the first-line medication for T2D and a key regulator of metabolism. Activation of adenosine 5'-monophosphate-activated protein kinase (AMPK) by metformin can activate neutrophils and regulate the secretion of cytokines with anti-inflammatory and antibacterial effects^9^. In diabetic mice, pretreatment with metformin could modify glucose flux across the airway epithelium and limit hyperglycemia-induced bacterial growth^10^. Therefore, this study aimed to compare the risks of pneumonia and respiratory death between metformin users and nonusers.

## Results

### Participants

**Figure 1:**
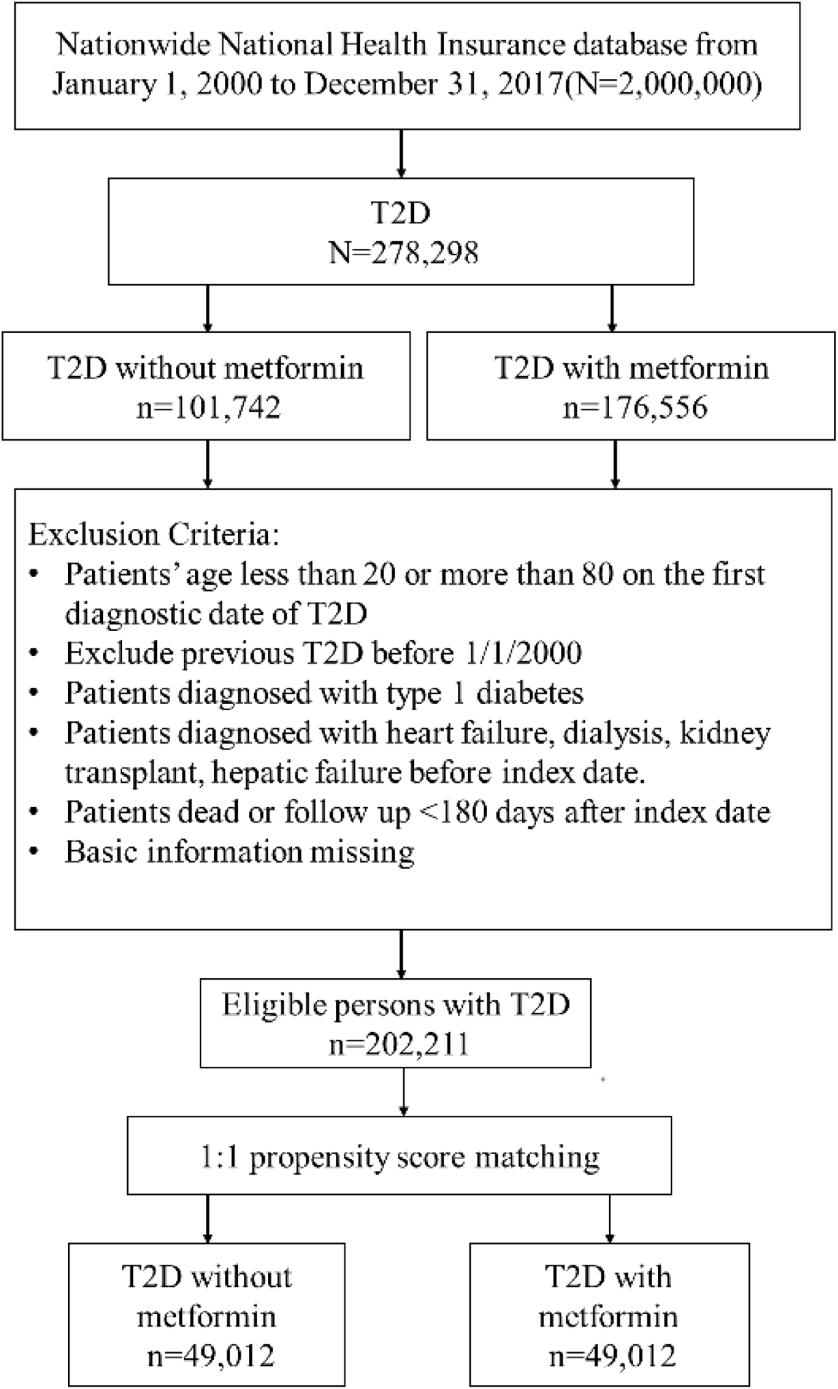
Flow diagram of the identified process.

From January 1, 2000, to December 31, 2017, we identified 278,298 patients with newly diagnosed T2D. Of these, 176,556 were metformin users, and 101,742 were nonusers (Fig. 1). After excluding ineligible cases, 1:1 propensity score matching was used to construct 49012 pairs of metformin users and nonusers. In the matched cohorts (Table 1), 54.16% of patients were female; the mean (SD) age was 57.46 (12.88) years. The mean follow-up time for metformin users and nonusers was 5.47 (3.71) and 5.15 (3.87) years, respectively.

**Table 1:** Comparison of characteristics between matched metformin users and nonusers in patients with T2D.

Variables	T2D without metformin		T2D with metformin		*P* value
	(N = 49,012)		(N = 49,012)		
	n	%	n	%	
**Sex**					0.279
Female	26,630	54.33	26,461	53.99	
Male	22,382	45.67	22,551	46.01	
**Age**					0.423
20–40	5436	11.09	5545	11.31	
41–60	22,166	45.23	22,215	45.33	
61–80	21,410	43.68	21,252	43.36	
Mean, (SD)^a^	57.53	12.88	57.39	12.87	0.095
**Comorbidities**					
Hypertension	27,240	55.58	28,422	57.99	< 0.0001
Dyslipidemia	29,244	59.67	29,799	60.80	< 0.0001
Coronary artery disease	14,212	29.00	14,128	28.83	0.554
Stroke	4361	8.90	4362	8.90	0.991
Atrial fibrillation	27	0.06	25	0.05	0.781
PAOD	1933	3.94	1935	3.95	0.974
CKD	3549	7.24	3491	7.12	0.473
Retinopathy	3766	7.68	3701	7.55	0.434
COPD	13,046	26.62	13,149	26.83	0.457
Liver cirrhosis	18,135	37.00	18,381	37.50	0.104
Cancers	2811	5.74	2777	5.67	0.640
Psychosis	115	0.23	104	0.21	0.457
Depression	17,717	36.15	17,380	35.46	0.025
Dementia	1345	2.74	1250	2.55	0.059
**CCI**					< 0.0001
1	9854	20.11	9346	19.07	
2–3	23,936	48.84	24,948	50.90	
> 3	15,222	31.06	14,718	30.03	
**DCSI**					0.023
0	17,411	35.52	17,072	34.83	
1	8751	17.85	9021	18.41	
≥ 2	22,850	46.62	22,919	46.76	
**Medications**					
SU	5223	10.66	5359	10.93	0.162
TZD	503	1.03	530	1.08	0.398
DPP-4i	444	0.91	480	0.98	0.234
AGI	1155	2.36	1209	2.47	0.261
**OAD drugs**					0.378
1	48,177	98.30	48,134	98.21	
2–3	820	1.67	867	1.77	
> 3	15	0.03	11	0.02	
Insulin	18,256	37.25	18,416	37.57	0.291
Statin	15,329	31.28	15,366	31.35	0.799
Aspirin	18,042	36.81	18,239	37.21	0.193

*PAOD* peripheral arterial occlusive disease, *CKD* chronic kidney disease, *COPD* chronic obstructive pulmonary disease, *CCI* Charlson comorbidity index, *DCSI* diabetes complication severity index, *SU* sulfonylurea, *TZD* thiazolidinedione, *DPP-4i* dipeptidyl peptidase-4 inhibitor, *AGI* alpha-glucosidase inhibitor.

Data shown as n (%) or mean ± SD.

^a^Student’s t-test. A *P* value > 0.05 indicates a negligible difference.

### Main outcomes

In the matched cohorts (Table 2), 2133 (4.35%) metformin users and 2220 (4.53%) nonusers developed bacterial pneumonia during the follow-up period (incidence rate: 7.86 vs. 8.72 per 1000 person-years). In the multivariable model, metformin users showed a significantly lower risk of bacterial pneumonia than nonusers (aHR = 0.89, 95% CI = 0.84–0.94). Compared with nonusers, metformin users also showed significantly lower risks of IMV (aHR 0.77, 95% CI 0.73–0.82) and respiratory cause of death (aHR 0.64, 95% CI 0.56–0.74); however, metformin users demonstrated insignificant risks for all-cause pneumonia (aHR 1.01, 95% CI 0.96–1.06) and NIPPV (aHR 0.93, 95% CI 0.86–1.01) (Table 2).

**Table 2:** Hazard ratios and 95% confidence intervals for outcomes in patients with metformin use versus nonuse.

Outcome	T2D without metformin			T2D with metformin			cHR	(95% CI)	*P* value	aHR^a^	(95% CI)	*P* value
	n	PY	IR	n	PY	IR						
All-cause pneumonia	2950	252,412	11.69	3203	268,320	11.94	1.02	(0.97, 1.07)	0.497	1.01	(0.96, 1.06)	0.793
Bacterial pneumonia	2220	254,691	8.72	2133	271,465	7.86	0.9	(0.84, 0.95)	< 0.001	0.89	(0.84, 0.94)	< 0.001
NIPPV	1211	256,617	4.72	1250	274,214	4.56	0.96	(0.89, 1.04)	0.332	0.93	(0.86, 1.01)	0.083
IMV	2382	256,847	9.27	1979	274,261	7.22	0.77	(0.73, 0.82)	< 0.001	0.77	(0.73, 0.82)	< 0.001
Respiratory causes of death	461	259,066	1.78	319	277,433	1.15	0.65	(0.56, 0.75)	< 0.001	0.64	(0.56, 0.74)	< 0.001

*NIPPV* noninvasive positive pressure ventilation, *IMV* invasive mechanical ventilation, *PY* person-years, *IR* incidence rate, per 1000 person-years, *cHR* crude hazard ratio, *aHR* adjusted hazard ratio.

*aHR*^a^, multivariable analysis adjusted for sex, age, comorbidities, CCI, DCSI scores, insulin, statin, aspirin, item and number of oral antidiabetic drugs.

**Figure 2:**
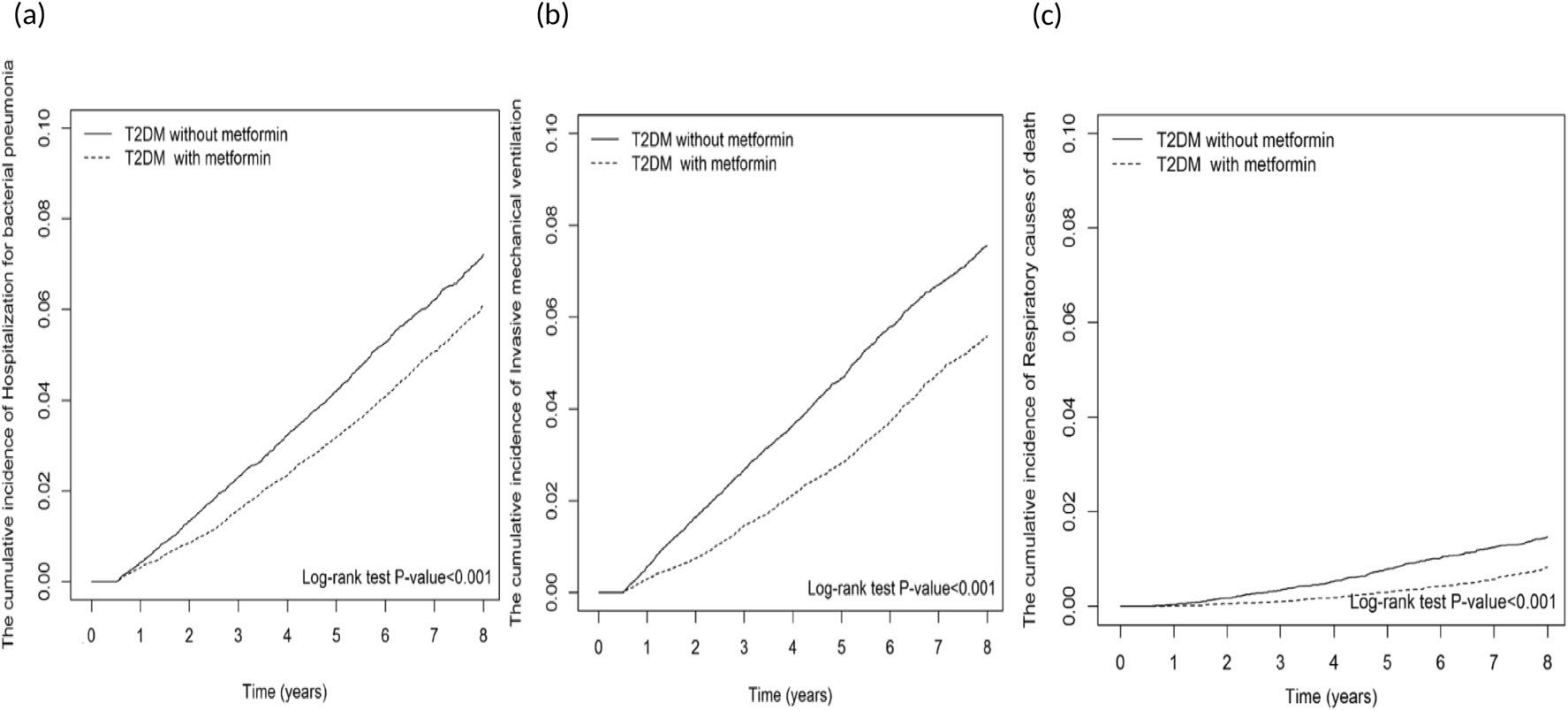
Cumulative incidences of outcomes between metformin users and nonusers. (**a**) Bacterial pneumonia, (**b**) invasive mechanical ventilation, and (**c**) respiratory causes of death.

The Kaplan–Meier analysis showed that the cumulative incidences of hospitalized bacterial pneumonia, IMV use, and respiratory cause of death were significantly lower in metformin users than in nonusers (Fig. 2).

### Cumulative duration of metformin use

We investigated the association between the cumulative duration of metformin use and the risks of bacterial pneumonia, IMV, and respiratory cause of death (Table 3). We observed that a longer cumulative duration of metformin use was associated with lower risks of bacterial pneumonia, IMV, and respiratory cause of death, and if metformin use exceeded 364 days, all *P* values of lower aHR in these three outcomes were less than 0.001 (Table 3).

**Table 3:** Hazard ratios and 95% confidence intervals for outcomes with the cumulative duration of metformin use.

Variables	Outcome			cHR	(95% CI)	aHR^a^	(95% CI)
	N	PY	IR				
**Bacterial pneumonia**							
Nonuse of metformin	2220	254,691	8.72	1.00	(Reference)	1.00	(Reference)
**Metformin of drug days**							
< 182	473	46,989	10.07	1.24	(1.13, 1.37)***	1.11	(1, 1.22)*
182–364	234	30,268	7.73	1.01	(0.88, 1.16)	1.01	(0.88, 1.15)
> 364	1426	194,209	7.34	0.81	(0.75, 0.86)***	0.82	(0.76, 0.87)***
**Invasive mechanical ventilation**							
Nonuse of metformin	2382	256,847	9.27	1.00	(Reference)	1.00	(Reference)
**Metformin of drug days**							
< 182	518	47,392	10.93	1.26	(1.14, 1.38)***	1.14	(1.04, 1.26)**
182–364	288	30,417	9.47	1.14	(1.01, 1.29)*	1.13	(1, 1.28)*
> 364	1173	196,453	5.97	0.62	(0.58, 0.66)***	0.63	(0.58, 0.67)***
**Respiratory causes of death**							
Nonuse of metformin	461	259,066	1.78	1.00	(Reference)	1.00	(Reference)
**Metformin of drug days**							
< 182	84	48,028	1.75	1.15	(0.91, 1.45)	0.95	(0.75, 1.2)
182–364	36	30,723	1.17	0.89	(0.63, 1.25)	0.88	(0.63, 1.24)
> 364	199	198,682	1	0.52	(0.44, 0.62)***	0.54	(0.46, 0.64)***

*PY* person-years, *IR* incidence rate, per 1000 person-years, *cHR* crude hazard ratio, *aHR* adjusted hazard ratio.

*P < .05, **P < .01, ***P < .001.

aHR^a^, multivariable analysis adjusted for sex, age, comorbidities, CCI, DCSI scores, insulin, statin, aspirin, item and number of oral antidiabetic drugs.

### Additional analyses

The active comparison of metformin versus sulfonylureas disclosed that metformin use was associated with a lower risk of bacterial pneumonia (aHR 0.65, 95% CI 0.59–0.73. Table 4). The time-varying analysis of metformin exposure showed that metformin use was associated with reduced risk of bacterial pneumonia (aHR 0.92, 95% CI 0.855–0.988. *P* = 0.0213. Table 4). The subgroup analysis of three age groups of 20–40, 41–60, and 61–80 years revealed that metformin use was associated with a higher risk of bacterial pneumonia in patients with age of 20–40 years (aHR1.81, 95% CI 1.28–2.57), and with a lower risk of bacterial pneumonia in patients with age of 61–80 years (aHR 0.84, 95% 0.78–0.9). The stratified analysis of four different metformin daily dosage displayed that the higher daily dose of metformin use was associated with lower risks of bacterial pneumonia compared with metformin no-use (Table 4). The comparison of metformin use versus no-use exhibited that metformin was not significantly different in the risk of COPD exacerbation (aHR 0.97, 95% CI 0.92–1.02) in patients with T2D and COPD (Table 4).

**Table 4:** Additional analyses for outcomes in patients with metformin use versus no-use or sulfonylurea.

Variables	Outcome			cHR	(95% CI)	aHR^a^	(95% CI)
	N	PY	IR				
**Bacterial pneumonia**							
Active comparator anaylsis							
Sulfonylurea use	862	122,874	7.02	1.00	(Reference)	1.00	(Reference)
Metformin use	592	124,602	4.75	0.68	(0.61, 0.75)***	0.65	(0.59, 0.73)***
**Time-varying exposure of metfomin**							
Nonuse of metformin				1.00	(Reference)	1.00	(Reference)
Metformin use				0.9	(0.838,0.967)**	0.92	(0.855, 0.988)*
**Age groups of metfomin users**							
Nonuse of metformin	2046	246,785	8.29	1.00	(Reference)	1.00	(Reference)
**Age groups of metformin users**							
20–40	50	33,180	1.51	1.93	(1.37, 2.73)***	1.81	(1.28, 2.57)***
41–60	369	115,250	3.20	1.13	(0.99, 1.3)	1.09	(0.95, 1.26)
61–80	1627	98,356	16.54	0.8	(0.74, 0.86)***	0.84	(0.78, 0.9)***
**Cumulative dose of metfomin use**							
Nonuse of metformin	2046	246,785	8.29	1.00	(Reference)	1.00	(Reference)
**Dose of metfomin (mg/day)**							
< 400	689	80,598	8.55	1.15	(1.06, 1.26)**	1.09	(1, 1.19)
400–799	439	58,963	7.45	0.95	(0.86, 1.06)	0.98	(0.88, 1.08)
800–1199	319	46,291	6.89	0.80	(0.71, 0.9)***	0.81	(0.72, 0.91)***
≧ 1200	538	76,228	7.06	0.74	(0.67, 0.82)***	0.76	(0.69, 0.84)***
**Acute exacerbation of COPD**							
Nonuse of metformin	2611	204,119	12.79	1	(Reference)	1.00	(Reference)
Metformin use	2703	214,200	12.62	0.98	(0.93, 1.04)	0.97	(0.92, 1.02)

*PY* person-years, *IR* incidence rate, per 1000 person-years, *cHR* crude hazard ratio, *aHR* adjusted hazard ratio, *COPD* chronic obstructive pulmonary disease.

*P < .05, **P < .01, ***P < .001.

aHR^a^, multivariable analysis adjusted for sex, age, comorbidities, CCI, DCSI scores, insulin, statin, aspirin, item and number of oral antidiabetic drugs.

## Discussion

Our study showed that metformin use in persons with T2D was associated with significantly lower risks of bacterial pneumonia, IMV, and respiratory death than metformin nonuse. Moreover, a longer duration of metformin use tended to offer better protection against bacterial pneumonia and respiratory outcomes. Specifically, all the outcomes mentioned above had statistically significant protective effects when metformin use exceeded 364 days.

A study from the Emerging Risk Factors Collaboration revealed that cardiovascular disease and cancer were the main causes of death in persons with diabetes. However, infectious diseases [HR 2.39 (1.95–2.93)] and pneumonia [HR, 1.67 (1.45–1.92)] also accounted for a higher risk of death compared to non-diabetes persons^11^. Specifically, pneumonia is the most important infectious disease in patients with T2D^3^. Preclinical studies demonstrated that sputum and bronchial aspirates were enriched with bacteria in animals with hyperglycemia^10^. Diabetes may narrow the capillary lumen of the lung and impair pulmonary function^6^. Thus, diabetes may be a unique risk factor associated with an increased incidence of pneumonia^1,4^ and higher risks of hospitalization and mortality due to pneumonia than the non-diabetic state^1,3^. The American Diabetes Association guidelines recommend that patients with diabetes receive anti-influenza and pneumococcal vaccines to decrease the risks of influenza and pneumonia^5^. However, coverage rates for vaccines are inadequate, especially pneumococcal vaccination^12^. More efforts directed to prevent pneumonia are urgently needed to reduce the risk of progression to respiratory failure and death.

Studies show that metformin can prevent and mitigate tuberculosis infection^13^. A study on mice with diabetes showed that metformin could limit hyperglycemia-induced *S. aureus* growth in the airway^10^. One nested case-control study found no differences in the incidence of community-acquired pneumonia between oral antidiabetic drugs in monotherapy and metformin in patients with T2D^14^. However, a Danish population-based cohort study showed a lower risk of pneumonia hospitalization in patients with T2D and initiation with metformin therapy versus sulfonylurea or insulin^15^. Subgroup analyses conducted in the two studies mentioned above assessed the impact of metformin use in pneumonia risk. Yang et al. conducted a retrospective cohort study, aiming to compare the risk of pneumonia between metformin use and nonuse in individuals with T2D from the Hong Kong Diabetes Registry. This study disclosed that long-term use of metformin was associated with reduced risks of pneumonia and pneumonia-related death^16^. Our study is consistent with Yang’s study, showing that metformin use was associated with an 11% lower risk of bacterial pneumonia, and a longer duration and a higher dose of metformin use tended to lower the risk of bacterial pneumonia further. The comparative analysis of metformin versus sulfonylurea, and the time-varying analysis of metformin exposure also demonstrated that metformin use was associated with a lower risk of bacterial pneumonia. Additionally, the three age groups analysis revealed that younger metformin users (20–40 years) were associated with a higher risk of pneumonia (aHR1.81, 95% CI 1.28–2.57), probably because the number of younger metformin users was relatively small, and they may have suboptimal compliance of clinic visits and taking medications. However, metformin use seems to have a protective effect against pneumonia in older patients. For the older people (61–80 years), metformin users were less likely to suffer from bacterial pneumonia (aHR 0.84, 95% 0.78–0.9).

Several studies have suggested that preadmission metformin use may reduce mortality risk in patients with T2D and sepsis^15^. Experimental studies have demonstrated that metformin can alleviate acute lung injury and various sepsis-induced organ injuries^17^. Our study showed that metformin use was associated with a 23% lower risk for invasive mechanical ventilation than metformin nonuse in persons with T2D. Although metformin use in patients with hypoxia or critical condition may cause lactic acidosis^17^, these studies suggest that metformin use may have beneficial effects in such patients.

Metformin use was associated with significantly lower mortality risk in women with obesity or T2D who underwent hospitalization for COVID-19 infection^18^. Our study also showed that metformin use was associated with a 36% lower risk of respiratory causes of death than metformin nonuse in patients with T2D. This study has shown that metformin use was associated with lower risks of bacterial pneumonia and IMV use, contributing to the lower risk of respiratory death. In brief, metformin may reduce the risk of death from respiratory causes in patients with T2D.

The possible explanations for the role of metformin in preventing the development and progression of bacterial pneumonia are as follows: (1) Metformin inhibits mitochondrial respiratory-chain complex-1 and activates the liver kinase B1 (LKB1)/ AMPK pathway to facilitate neutrophil-dependent bacterial uptake and killing, and promote innate immune response^18^. (2) But it can suppress pro-inflammatory markers of high sensitivity C-reactive protein, interferon-α(IFN-α)^19^, tumor necrosis factor-α (TNF-α), and interleukin -6 (IL-6); and inhibits neutrophil activation and chemotaxis, improves neutrophil to lymphocyte ratio^18^, reduces B-cell intrinsic inflammation, increases antibody response, and stabilizes mast cells^18^. Metformin also can boost levels of the anti-inflammatory marker IL-10^18^. (3) The inhibition of mitochondrial complex-1 and electron transport can also suppress the energy production required for bacterial growth. Metformin inhibits bacterial gluconeogenesis, and the limited utilization of glycerol in the Kreb’s cycle reduces bacterial virulence; the anti-folate effect of metformin may inhibit the folate cycle of bacteria and limit bacterial growth^20^. Thus, metformin may attenuate the risk of bacterial pneumonia by its metabolic, immunologic, and antibacterial effects.

This study had some limitations. First, this dataset lacked information on family history, dietary patterns, physical activity, alcohol use, smoking habits, and vaccination status; it did not include data on hemoglobin A1C, biochemical tests, renal function, immune status, and pulmonary function test, precluding an accurate understanding of patient health status, and the severity of T2D. However, we matched the demographic information on age and sex to achieve a balance between the study and control groups; we matched the type and number of oral antidiabetic drugs, insulin use, and DCSI scores to balance the severity of T2D; we have matched the comorbidity of CKD to decrease the influence of renal function on outcomes, and further increase the comparability between the study and control groups. Second, metformin is contraindicated in patients with eGFR < 30 ml/min/1.73 m^2^. Because the NHIRD dataset lacks the information of renal function, we only excluded patients on dialysis or kidney transplant to avoid the effects of confounding by indication. Third, this study was conducted on Taiwanese people, and the results may not be generalizable to other ethnicities. Finally, cohort studies are likely to be influenced by some unknown and unmeasured confounding factors, and randomized controlled studies are warranted to confirm our results.

Standards of diabetes care have gradually become more comprehensive. A longer life expectancy in patients with T2D may contribute to the rise of several non-communicable and communicable diseases needing vigilance. Specifically, pneumonia is a critical communicable complication in patients with T2D; however, there are few recommendations on preventing pneumonia. Our study demonstrated that metformin was associated with lower risks of bacterial pneumonia, IMV use, and respiratory death. Metformin can play a beneficial role in reducing the risk and progression of pneumonia.

## Methods

### Study population

The Taiwanese government formed the Bureau of National Health Insurance in 1995 to establish the National Health Insurance (NHI) program. This program is a compulsory insurance system. Until 2000, nearly 99% of Taiwan’s 23 million people have joined the NHI program^21^. All information of the insured people, including age, sex, residence, insurance premium, diagnosis, medical procedures, and medications, are recorded in the NHI Research Database (NHIRD). The diagnosis is based on the International Classification of Diseases, Ninth Revision, Clinical Modification (ICD-9-CM). The NHIRD is linked to the National Death Registry to certify mortality information. We confirmed that all methods were performed in accordance to Declaration of Helsinki. This study was approved by the Research Ethics Committee of China Medical University and Hospital (CMUH109-109-REC2-031). The identifiable information of patients and caregivers was encrypted before release, and informed consent was waived by the Research Ethics Committee.

### Study design

We identified patients newly diagnosed with T2D between January 1, 2000, and December 31, 2017, and followed them up until December 31, 2018. The diagnosis of T2D was based on the ICD-9-CM code 250.xx for at least 2 outpatient visits or one hospitalization record. A previous study in Taiwan has performed a validation using ICD codes to define T2D^22^. Patients were excluded (Fig. 1) if they were (1) younger than 20 or older than 80 years; (2) missing age or sex data; (3) diagnosed with type 1 diabetes (250.1x) or heart failure (398.91, 402.01, 402.11, 402.91, 404.01, 404.03, 404.11, 404.13, 404.91, 404.93, 428, 429.4), received dialysis (V56.0, V56.8, V45.1) or kidney transplant (V42.0x), had hepatic failure (570, 572.2, 572.4, 572.8); (4) diagnosed with T2D before January 1, 2000, to exclude prevalent diseases before 2000; (5) followed up for less than 6 months after the index date.

### Procedures

We defined the date of metformin use as the index date. Patients who received metformin treatment ≧ 28 days were study cases and those who never received metformin served as controls. The index date of the metformin nonusers was assigned as the same date as their corresponding paired metformin users’ index date. Some related variates were assessed and matched between the metformin user and nonuser, including age, sex, comorbidities included hypertension (401–405 and A26), dyslipidemia (272), coronary artery disease (CAD; 410–414), stroke (430–438), atrial fibrillation (427), peripheral arterial occlusive disease (PAOD; 440.0, 440.20, 440.21, 440.22, 440.23, 440.24, 440.3, 440.4, 443.9, 443.81, 443.89), chronic kidney disease (250.4x, 403.xx, 404.xx, 585.xx, 586.xx, 581.8x, 791.0x, 593.9x), retinopathy (362.02, 362.07, 362.0), chronic obstructive pulmonary disease (COPD; 491, 492, 496), liver cirrhosis (571.5, 571.2, 571.6), cancers (140–239), psychosis (290–299), depression (311), and dementia (290, 290.4, 291.2, 292.82 and 331), diagnosed within 1 year before the index date; medications use, including oral antidiabetic drugs, insulin, statin, and aspirin, during the follow-up period. We used the Charlson Comorbidity Index (CCI), Diabetes Complication Severity Index (DCSI) score^23,24^, and the number of oral antidiabetic drugs to evaluate T2D severity.

### Main outcomes

Hospitalization for all-cause pneumonia (480–486), bacterial pneumonia (481, 482.41, 482.8, 486), noninvasive positive pressure ventilation (NIPPV; 93.90 and 93.91), invasive mechanical ventilation (IMV; 96.7) use, and respiratory causes of death (460–466, 470–478, 480–488, 490–496, 500–508 and 510–519) were the main outcomes of this study. A previous study in Taiwan has validated the algorithm of using ICD-9 codes to define pneumonia, with a sensitivity of 92.3–94.7%^25^. We calculated the events and incidence rates for hospitalized all-cause pneumonia, bacterial pneumonia, NIPPV, IMV, and respiratory causes of death during the follow-up period. The cumulative incidences of bacterial pneumonia, IMV, and respiratory causes of death were also compared between metformin users and nonusers.

### Statistical analysis

Propensity-score matching was used to optimize the related covariates between metformin users and nonusers^26^. We estimated the propensity score for each patient using non-parsimonious multivariable logistic regression, with metformin use as the dependent variable. We included 28 clinically relevant covariates as independent variables (Table 1). The nearest-neighbor algorithm was used to construct matched pairs, assuming the *P* value > 0.05 to be a negligible difference between the case and comparison cohorts.

Crude and multivariable-adjusted Cox proportional hazards models were used to compare outcomes between metformin users and nonusers. The results are presented as hazard ratios (HRs) and 95% confidence intervals (CIs) for metformin users compared with nonusers. To calculate the investigated risks, we censored patients on the date of death, date of respective outcomes, or at the end of the follow-up on December 31, 2018, whichever came first. The Kaplan–Meier method and log-rank tests were used to compare the cumulative incidence of bacterial pneumonia, IMV, and respiratory cause of death during the follow-up period between metformin users and nonusers. We also assessed the cumulative duration of metformin use for the risks of bacterial pneumonia, IMV, and respiratory cause of death compared with metformin nonuse.

We have performed some additional analyses. (1) The comparative analysis of metformin versus sulfonylureas by matching demographics, comorbidities, medication use and disease stage, to provide an active comparison of metformin against sulfonylurea in the risk of bacterial pneumonia. (2) A time-varying analysis, assigning metformin exposure as a time-varying covariate, to decrease the biases of metformin discontinuation or immortal time bias. (3) A subgroup analysis of three age groups of 20–40, 41–60, and 61–80 year to investigate different effects of metformin use in different age groups. (4) A stratified analysis of four different metformin daily dosage to detect dose-response effects for metformin use. (5) A sensitive analysis to compare the incidence rates of acute COPD exacerbation (indicated by the prescription of systemic corticosteroids or antibiotics at outpatient department, hospitalization or an emergency room visit for COPD) of metformin use versus metformin no-use in patients with T2D and COPD.

SAS (version 9.4; SAS Institute, Cary, NC, USA) was used for statistical analysis; a two-tailed *P* value < 0.05 was considered significant.
